# Associations between Biological Maturation, Chronological Age, Body Mass Index, Sex, and Motor Competence in Prepubertal Children: A Network Analysis

**DOI:** 10.3390/children11091143

**Published:** 2024-09-20

**Authors:** Sedigheh Salami, Paulo Felipe Ribeiro Bandeira, Maryam Rahymian Mashhadi, Parvaneh Shamsipour Dehkordi, Leonardo G. O. Luz, Clarice Maria de Lucena Martins, Louise L. Hardy, Michael J. Duncan

**Affiliations:** 1 Department of Motor Behavior, Faculty of Sport Sciences, Alzahra University, Tehran 15847-15414, Iran; sed60sal@gmail.com (S.S.); m.rahimian@alzahra.ac.ir (M.R.M.); p.shamsipour@alzahra.ac.ir (P.S.D.); 2 GEAPAM—Department of Physical Education, Regional University of Cariri, Crato 63105-010, Brazil; paulo.bandeira@urca.br; 3 LACAPS, Campus Arapiraca, Federal University of Alagoas, Arapiraca 57309-005, Brazil; leonardoluz@arapiraca.ufal.br; 4 CIDAF, Faculty of Sport Science and Physical Education, University of Coimbra, 3004-531 Coimbra, Portugal; 5 Departamento de Educação Física, Universidade Federal da Paraíba, João Pessoa 58051-900, Brazil; clarice@fade.up.pt; 6 Laboratory for Integrative and Transitional Research in Population Health, Research Centre in Physical Activity, Health and Leisure, University of Porto, 4200-450 Porto, Portugal; 7 Prevention Research Collaboration, School of Public Health, University of Sydney, Sydney, NSW 2006, Australia; louise.hardy@sydney.edu.au; 8 Centre for Physical Activity, Sport and Exercise Sciences, Coventry University, Coventry CV1 5FB, UK

**Keywords:** complex systems, children, TGMD-3, motor competence

## Abstract

Background: The development of motor competence (MC) during childhood is crucial for future physical activity and health outcomes, and it is affected by both biological and psychosocial factors. Most MC research has focused on children’s age, with fewer studies examining separate associations between MC and biological maturation. Methods: This cross-sectional study used network analysis to assess the nonlinear associations between biological maturation (the child’s percentage of predicted mature stature to indicate somatic maturation), chronological age, sex, BMI, and MC (Test of Gross Motor Development, third edition) in 218 children (100 boys, 118 girls) aged 7–9 years. Results: Biological maturation was not significantly associated with MC in boys and weakly associated with MC in girls for the dribble, under-hand throw, and gallop. Age was positively associated with MC in girls and boys. Centrality measures indicated that the gallop and slide in girls and the dribble, catch, and run in boys were the most important network variables. Positive associations were observed between maturation and BMI for girls (r = 0.579) and, to a lesser degree, for boys (r = 0.267). Conclusions: The findings suggest that age, rather than biological maturation, is positively associated with MC in 7- to 9-year-olds. Centrality measures showed that some skills may influence other skills.

## 1. Introduction

Motor competence (MC) describes a person’s capability to perform a wide range of motor movements, including locomotor, object control, and stability skills [1]. In children, MC is associated with engagement in physical activity, perceived MC, and maintaining higher levels of physical activity and cardiorespiratory fitness in adolescence and adulthood [2,3,4]. Several interacting factors can influence MC development in childhood, including psychology, the social environment (e.g., gender roles, parenting style, stereotypes, experiences, play opportunities, and motivation) and biology (e.g., age, genetics, sex, and maturation) [5,6]. Previous research has suggested a potential inter-relationship between biological maturation, growth, and MC [1]. However, most MC research has focused on children’s chronological age, with fewer studies examining any separate associations between MC and biological maturation. Therefore, the nature of these associations may be more complex and nonlinear, warranting further investigation.

Biological maturation is a measure of maturational status, timing, and tempo [7]. Status pertains to the state of maturation at the time of observation, timing pertains to the age when particular maturational events, such as menarche, take place, and tempo refers to the rate at which maturation progresses [7]. In some children, early maturation may commence prior to the age of eight for girls and nine for boys [8].

Skeletal age is a common indicator of biological maturation but involves radiation exposure; hence, noninvasive methods for estimating biological maturation are frequently utilized in field studies [9]. Such measures include the percentage of predicted mature (adult) stature attained at the time of measurement and the predicted maturity offset (time before or after peak height velocity (PHV)/age at PHV). The former offers an estimate of maturity status, whereas the latter provides an estimate of the timing of maturity [10]. The accuracy and reliability of the age at PHV and predicted maturity offset have been questioned [11]. An alternative indicator suitable for young children is the Khamis and Roche method (1994) [12], which is determined by the percentage of predicted adult height attained at the time of assessment.

Previous researchers have examined associations between indicators of biological maturation and MC by applying different analytical approaches and highlighting several interactions [13,14,15]. For example, applying hierarchical multiple regression, skeletal age alone, and in interaction with body size, was only marginally related to MC in Portuguese youth aged 7–10 years and 11–14 years [13,16]. A canonical correlation analysis found only weak associations between MC tasks (measured by the KTK battery) and biological maturation [15]. Most existing studies examined associations among isolated constructs using regression models to examine linear relationships between biological maturation, growth, and MC tasks. These approaches disregard the synergic, dynamic, and nonlinear interactions between these variables. Considering the multifactorial nature of MC, alternative analytical methods are necessary to enhance our understanding of the interactions among biological variables and MC.

Network analysis provides an alternative method to inferential statistics for predicting multidimensional nonlinear relationships among variables [17,18]. In contrast to linear models like regression, a network analysis approach uses centrality measures such as closeness, betweenness, strength, and expected influence of variables to evaluate and visualize nonlinear and complex interactions. That is, it can identify developing patterns and significant variables that could influence nonlinear complex behaviors such as MC [19,20].

We hypothesized that the associations between biological maturation, chronological age, body mass index (BMI), sex, and MC comprise a nonlinear network and these variables interact with each other to create a developing nonlinear pattern that identifies the key associative variables within the network. To mitigate the impact of chronological age as a source of inter-individual variability, we selected prepubertal children aged 7 to 9 years for this research [10]. We used network analysis to explore the nonlinear associations among maturation, age, sex, BMI, and MC to identify which are the most salient factors of influence in the development of MC in 7–9-year-old children.

## 2. Materials and Methods

### 2.1. Study Design

This was a cross-sectional study of prepubertal Iranian school children conducted in February–March 2017. Eligible children were enrolled in school, aged between 7 and 9 years old, without perceptual impairments (such as visual or auditory deficits), and without any documented history of physical, intellectual, or neurological disorders. Parents/legal guardians were provided with a letter explaining the objectives and procedures of the study, including a declaration of confidentiality, voluntary participation, and the child’s right to withdraw from the study at any time. Signed informed consent from the parents was required in order for children to participate. The research protocol for this study received approval from the Ethics Committee of the Sport Sciences Research Institute of Iran (IR. SSRI. REC. 1399.728) and was developed in line with the principles established in the Declaration of Helsinki.

### 2.2. Participants

A convenience sample of 218 children, aged 7 to 9 years (*M* age = 8.0, *SD* = 0.96; 54.1% girls), from four public primary schools in the Southeastern District of Tehran, Iran, were recruited. Schools were provided with a description of the study. Iranian primary schools deliver a specific curriculum between 7:30 am and 12:30 pm. Children have access to the playground for 30 min daily and one 60 min PE class per week.

### 2.3. Measurements

Motor Competence Assessment. Children’s MC was assessed by the Test for Gross Motor Development Edition 3 (TGMD-3) [21], a validated, process-orientated measure of MC that focuses on fundamental movement skills (FMSs) in children aged 3–10 years. Metric properties of the TGMD-3 showed excellent internal item consistency (α = 0.97) and test–retest reliability values for locomotor skills, object control, and total TGMD-3 (r > 0.95). The TGMD-3 assesses thirteen FMSs, six locomotor skills (run, skip, slide, gallop, hop, and horizontal jump), and seven object control skills (over-hand throw, under-hand throw, catch, dribble, kick, one-hand strike, and two-hand strike). The maximal locomotor and object control subtest scores are 46 and 54, respectively, and the maximum total TGMD-3 score is 100.

Two assessors (human movement students) were trained on the administration and protocol of the TMGD-3 [22] one week before the commencement of data collection for the testing protocol. The TGMD-3 was administered by them and the testing time was approximately 20–25 min per child. The TGMD-3 assessments were conducted in each school’s playground, and all evaluations took place during school hours, with physical education teachers present. All equipment was arranged in advance, and a comprehensive demonstration along with verbal instructions for each skill was provided at the beginning of each section of the test. All skills were video recorded for later analysis. Children were assessed individually and completed one practice attempt before performing the skill across two trials, which were scored according to the established performance criteria (0 = did not perform correctly; 1 = performed correctly). Higher scores on the TGMD-3 reflected a higher degree of MC.

The assessors independently evaluated the performance of a subsample (n = 47) for the inter-rater reliability. For intra-rater reliability, 80 assessments were analyzed twice by one assessor within a one-month interval. The inter- and intra-rater reliability were measured using intraclass correlation coefficients (ICCs). The reliability coefficient for inter-rater (range, 0.90 to 0.95) and intra-rater reliability (range, 0.85 to 0.90) indicated strong and congruent results among the assessors [23]. After confirming the intra- and inter-rater reliability, the first rater coded 60% of the participants and the second rater coded the remaining 40%.

Body Mass Index. Children’s height (in centimeters) and weight (in kilograms) were measured prior to assessing MC, in private, while they were dressed in light clothing and were barefoot. Height was measured with a stadiometer (Harpenden model 98.603, Holtain Ltd., Crosswell, UK) to the nearest 0.1 centimeters (cm). Weight was measured with a portable scale (Seca model 770, Hanover, MD, USA) to the nearest 0.1 kilograms (kg). Body mass index (BMI) was then calculated (weight (kg)/height (m^2^)).

Biological Maturation. The percentage of predicted mature stature (%PMS) was calculated using the Khamis and Roche method (1994) [12] based on the child’s current age (to one decimal point), height, and the average stature of the child’s parents. Children’s chronological age was calculated as the difference between children’s date of birth and the date of measurement. Parents were asked to self-report their height (in cm) and weight (in kg) when they signed the study consent form. All parents reported their height. As there is a tendency for adults to overestimate their height [24], the self-reported height of each parent was adjusted for overestimation using Epstein’s sex-specific equation. The equations and correlation coefficients for the height equations (y = adjusted value and x = self-reported measurement) for women were y = 2.803 + 0.953x, r = 0.977 and for men were y = 2.316 + 0.955x, r = 0.952 [24].

Children of the same chronological age who are closer to their expected adult height are regarded as being more mature than their peers who are further away from their predicted height [7]. The mean (median absolute deviation) and 90% error bounds between the actual and predicted mature stature (PMS) for children aged 4 and 18 years using the Khamis–Roche method are 2.2 and 5.3 cm, respectively, for boys and 1.7 and 4.3 cm, respectively, for girls [12]. Using PMS as the reference, the percentage of PMS (%PMS) for each child at the time of observation was determined in order to estimate biological maturation [12]. For every child, a z-score of %PMS was computed by comparing the child’s %PMS to the sex- and age-specific means and standard deviations derived from the Berkeley Guidance Study [25]. The %PMS value was calculated using the following formula:

%PMS (cm) = β0 + β1 height (cm) + β2 weight (kg) + β3 corrected mid-parent height (cm). The intercept (β0) and the coefficients (β1, β2, β3) in this equation depend upon the child’s age and sex [12].

### 2.4. Statistical Analysis

There were no missing data and data were checked for normality prior to the analysis. Descriptive statistics, including means and standard deviations, were calculated for the children’s variables, stratified by sex. Student’s *t*-test was employed to assess differences between sexes in chronological age, biological maturation indices, BMI, and MC tasks. The effect size of the differences between means was calculated via Cohen’s d values and values were interpreted as follows: <0.20 (trivial), 0.20 to 0.59 (small), 0.60 to 1.19 (moderate), 1.20 to 1.99 (large), 2.0 to 3.9 (very large), and >4.0 (extremely large) [26].

Network analysis was employed to examine the associations among biological maturation, chronological age, BMI, sex, and MC tasks for the total sample and by sex. This analytic method determines the associations between variables, considering the complexity and nonlinearity of the associations [20]. Correlations from a weighted matrix are used in the network to visually display connections through nodes and edges, where the nodes symbolize various variables and the edges denote the strength of connections between two or more nodes [18]. In the analysis, correlations are adjusted according to the nature and distribution of the variables. In this research, the nodes include biological maturation (PMS z-scores), chronological age, BMI, sex, and motor coordination skills, while the edges depict the positive and negative associations among these nodes. Blue edges signify positive associations, whereas red edges indicate negative associations. The thickness and intensity of the edges reflect the magnitude of these associations.

The significance of a node within a network is determined by its centrality, which reflects its influence or structural relevance, with higher associative values denoting greater significance [17]. To assess the centrality of nodes, three indices were used. The first was the betweenness index, which is calculated based on the frequency with which a node appears in the shortest paths connecting all pairs of nodes within the network. Nodes exhibiting higher betweenness values are deemed more susceptible to change and can function as hubs linking various pairs of variables in the network. The second was the closeness index, defined as the inverse of the average shortest distance from a node to all other nodes in the network. The third was the strength index, which represents the cumulative weights of all paths connecting a node to other nodes. The strength index is crucial to identifying which variables exhibit the most substantial connections within the network structure [27].

The centrality index calculates the expected influence, which reflects the importance of a node within the network’s structure and functionality. This measure is derived from the sum of all possible edge weights linking one node to another, facilitating an evaluation of the nature and intensity of a variable’s cumulative impact within the network as well as its anticipated role in the processes of activation, persistence, and remission. If some edges are negative, the centrality estimate for the node remains unchanged because the absolute values of the edge weights are summed. Positive expected influence values indicate that the nodes “turn on” the network (i.e., have a positive influence on other nodes), while negative values indicate that the nodes “turn off” the network (i.e., have a negative influence on other nodes) [28]. The centrality Plot function within the qgraph (version 1.1.463, Boston, MA, USA) software was used to calculate centrality indices for the overall group and differentiated by sex. Standardized z-scores were calculated for each index of centrality to allow for comparisons between networks with a mean of 0 and a standard deviation of 1, where an index value of >1 indicated that it was >1 *SD* from the mean. The further the index is from zero, the greater the relevance of the variable within the network.

In order to compute and visualize the network, we applied the Fruchterman–Reingold algorithm in which the data show the relative space between the variables such that the highest associations remain together and the weakest associations are pushed apart [29]. To enhance the accuracy of the network, we used the “random fields of pair-wise Markov” model, with the accuracy being assessed through the “L1” algorithm, which is a form of regularized neighborhood regression. The regulation was determined using a less comprehensive selection and contraction operator known as LASSO, aimed at managing the sparse nature of the network [30]. To mitigate the occurrence of spurious associations, we applied the Extended Bayesian Information Criteria (EBIC) parameter [31]. The EBIC parameter was found to select the optimal Lambda for the regularization parameter, utilizing a tuning hyperparameter (y) that dictates the extent of the regularization or penalty imposed on sparse correlations. In this investigation, the parameter was established at 0.25 (with typical values ranging from 0 to 0.5), which is considered a more parsimonious choice for exploratory networks [31].

Network analysis is not inferential, so the sample size depends on the complexity of the model. When dealing with small datasets, the network analysis process applies the Least Absolute Shrinkage and Selection Operator (LASSO) to regularized algorithms to obtain the precision matrix. This methodology offers a holistic view of the interactions between variables. These data were analyzed with qgraph and ggplot2 from the R Studio (version 1.1.463, Boston, MA, USA) software.

## 3. Results

Descriptive statistics for chronological ages, PMS, %PMS, z-scores of %PMS, anthropometrics, and each MC skill are presented in Table 1. Boys showed higher values for PMS than girls and girls had higher %PMS values than boys. For MC tasks, boys demonstrated better performance, compared with girls, on the two-hand strike, one-hand strike, catch, and over-hand throw, and girls performed better than boys on the run.

**Figure 1:**
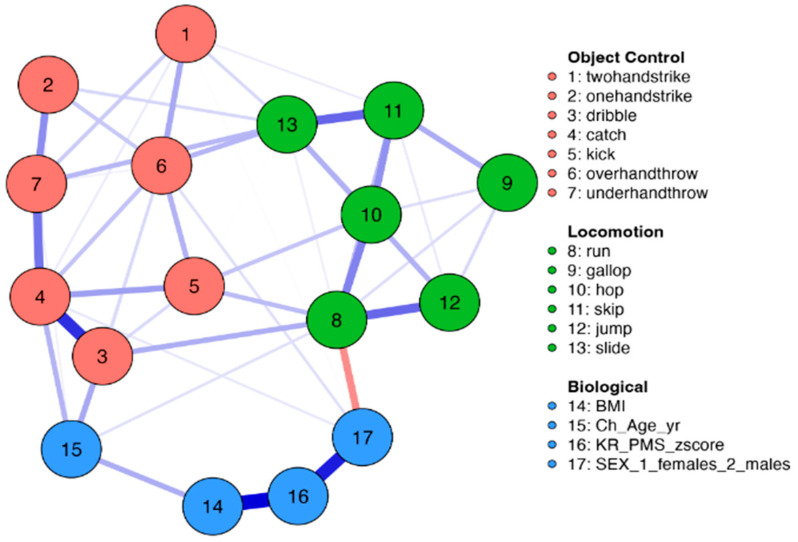
Network for the associations between domains for the total sample, biological variables, and object control and locomotion skills. Blue edge, positive association; red edge, negative association. The thickness of the lines indicates the weight of the ratio. Red node, object control skills; green node, locomotive skills; blue node, biological variables. BMI, body mass index; Ch-age-yr, chronological age; KR-PMS-z-score, Khamis and Roche predicted maturity status z-score.

**Figure 2:**
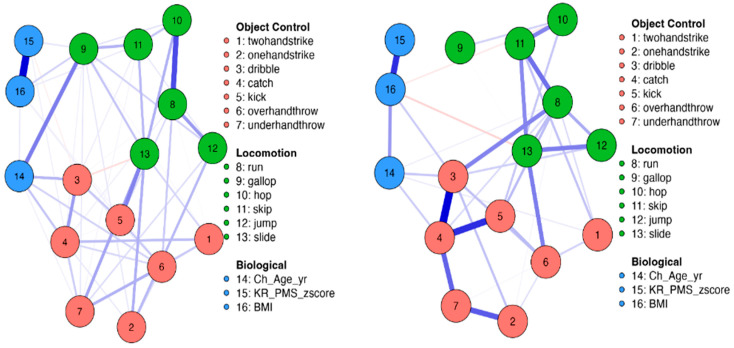
Network for the associations between domains by sex (girls and boys), biological variables, and object control and locomotion skills. Blue edge, positive association; red edge, negative association. The thickness of the lines indicates the weight of the ratio. Red node, object control skills; green node, locomotive skills; blue node, biological variables. BMI, body mass index; Ch-age-yr, chronological age; KR-PMS-z-score, Khamis and Roche predicted maturity status z-score.

The weights matrix of the network analysis is shown in Table 2 separately for girls and boys. Biological maturation (PMS z-scores) was strongly and positively related to BMI in girls (0.579) and moderately positive in boys (0.267). In boys, MC items were not associated with PMS z-scores; however, in girls, weak associations were observed between PMS z-scores and the dribble (−0.039), under-hand throw (0.038), and gallop (0.064). Age was positively associated with the dribble (0.169), catch (0.138), over-hand throw (0.108), under-hand throw (0.037), and gallop (0.297) in girls. In boys, weak positive associations were seen between age and dribble (0.074), catch (0.068), and run (0.022). BMI associations with MC items were weak and positive for the kick (0.042), over-hand throw (0.027), gallop (0.106), and skip (0.051) in girls. In boys, the associations with BMI were weak and negative for the hop (−0.033) and slide (−0.059). The network configurations of associations for the total sample and by sex are shown in Figure 1 and Figure 2, respectively.

The values for the network centrality indices in Table 3 show the role of each variable in the network for the total group and by sex. For the total sample and for girls, the gallop and slide had the highest centrality indices (i.e., betweenness, closeness, strength, and expected influence). In boys, the highest centrality indices were the dribble, catch, and run.

## 4. Discussion

This is the first study to apply network analysis to explore the associations between biological maturation, chronological age, sex, BMI, and MC in 7–9-year-old Iranian children. Our results indicate that age, rather than biological maturation, is associated with MC in children aged 7–9 years. This finding could be explained by variables not included in this study that are closely related to MC and age and not with biological maturation. Potentially, psychological and cultural factors may affect children’s engagement in motor skills. In practical terms, interconnected variables can interact in multifaceted ways, illustrating that age-related psychosocial and cultural factors may also contribute significantly to children’s motor skill development. The connection between age and MC is not straightforward; as children age, improvements in MC are not always consistent. Theories in ecology and development indicate that various factors, including peer relationships and parental involvement, influence children’s engagement in physical activities, and these factors can evolve throughout childhood. Thus, it could be beneficial to explore models that incorporate interactions and moderating influences among variables like sex, BMI, and psychosocial elements to gain a more nuanced understanding of their collective impact on MC [2,5]. Although not directly comparable, previous studies also found that biological maturation was not a significant predictor of children’s MC, regardless of the maturation assessment method [13,15,16]. The development of MC in the context of biological maturation has received less attention than the association between age and MC. The inter-individual variation in maturity status during the peri-pubertal stage may influence children’s performances on tests of MC [10] and therefore warrants examination.

Several researchers have used skeletal age as an indicator of biological maturation. A study of Belgian girls aged 6–16 years showed that skeletal age alone and skeletal age in combination with chronological age, height, and body mass were not significant predictors of the standing long jump, the vertical jump, and the shuttle run [32]. A study of Portuguese children aged 7–10 years also showed that skeletal age alone and bone age interacting with body mass were negligibly related to MC [13]. These authors noted that a standardized residual of skeletal age alone explained a maximum of 9% of the variance in MC over and above the effect of body mass per se and the effect of interactions between body mass and the standardized residual of skeletal age on chronological age.

Given the role of height in biological maturation, several studies have used the %PMS attained at the time of observation as a noninvasive indicator of biological maturity. Eaton and Yu (1989) [33] reported a negative association between %PMS and activity levels in U.S. children aged 5–9 years. Similarly, Luz et al. (2018) [15] indicated that the associations between %PMS, fitness, and MC were negligible in eight-year-old Brazilian girls. In Austria, a longitudinal study among children aged 10–11 years used age at PHV to assess associations between biological maturity and MC and concluded that the associations between biological maturation and MC were generally weak after adjusting for body weight [34]. These findings support ours and suggest that there is an insignificant association between biological maturation and MC in prepubertal children. Together, these findings suggest that other factors, such as neuromuscular maturation, opportunities to participate in movement, play activities, organized sports, quality of preschool programs and physical education, and peer interactions, may influence MC at this age.

We found a positive association between BMI and biological maturation and, in boys, with age. While both indices include height, higher body fat during childhood is an important correlate of earlier onset of maturation, particularly in girls [7]. In the present study, BMI showed weak associations with some locomotion tasks in both sexes. This concurs with a multi-country study of 3–5-year-old children that reported negative associations between BMI and locomotor skills, object control skills, and overall MC, which became stronger in children at >97th BMI percentile [35]. Potentially, obesity in children impacts MC because it impedes stabilization and/or propulsion of the body during weight-bearing activities. However, studies that have analyzed the association between BMI and MC in models that consider, in isolation, the association between each MC skill and BMI, as carried out in the present study, are limited. Furthermore, the current evidence on the association between object control skill tasks and BMI is equivocal due to an insufficient number of longitudinal studies [3].

Consistent with previous investigators [2,36,37], we found no significant sex differences in children’s locomotor skills, except the run. Potentially, this is because locomotor skills may be considered more phylogenetic than object control skills; running skills do not require specific equipment, and, generally, there are greater opportunities for children to run. In this study, boys performed better than girls in object control skills (two-handed strike, one-handed strike, and over-hand throw), which is consistent with other studies [2,38]. In Iran, boys are more physically active than girls, and boys are provided with more opportunities to participate in physical activities [39]. Additionally, in Iranian society, boys receive greater encouragement, support, and opportunities to engage in high-impact physical activities and sports at home, in school, and the broader community, while girls’ opportunities to improve their MC are limited across childhood and adolescence.

Our network analysis showed that the dribble, catch, and run skills in boys and the slide and gallop in girls were the most important network MC skills, indicating that these skills may have an influencing role in the development of children’s MC. The high betweenness indices indicate that these skills act as hubs, connecting other pairs of variables in the network. The high closeness indices show that these skills are more likely to quickly affect other skills. The high strength indices of these skills indicate their robustness, allowing for a spreading effect to the other skills. The high expected influence indices indicate the role these skills have in the activation, persistence, and remission of the network. In practical terms, these skills may be more sensitive to interventions and may easily influence other skills. The identification of key MC skills, and their centrality in the network, indicates the nuanced nature of MC skill interdependencies. The network analysis highlights that certain MC skills emerge as critical influencers, suggesting that interventions should not solely focus on these central skills but rather consider the interconnectedness of all skills within MC.

MC inherently requires high levels of neuromuscular coordination and control and synchronized movements of the legs and arms [40]. Potentially, for children who demonstrate greater variability in skill performance, this may increase the power of particular skills to differentiate their performance levels in relation to their overall MC. While the role of other MC skills in children’s overall health is well established, this network analysis suggests that, although each skill has a discriminating role across the whole network, the dribble, catch, and run in boys and the slide and gallop in girls had the most influence. Our findings support a similar study of the TGMD-3 short version using the same approach [41]. Based on centrality measures from that network analysis, Duncan et al. (2022) concluded that the bounce and catch had a higher strength value.

It is important to note that, although the network centrality measures emphasized that some skills may exert the most (positive) influence on other skills, this does not mean that we should focus only on those skills in intervention programs. Indirect effects of all the other skills in the network should be considered as well. Children are dynamic and ever-evolving, with different development domains changing at different rates due to both intrinsic and extrinsic factors. Thus, while the current results show similar network structures between boys and girls, it cannot identify the specific causes of the different centrality measures observed between sexes. Further studies that include cultural, social, and environmental factors that are correlated with children’s MC [3] are required to better understand the development of MC in boys and girls. Understanding the effects of cultural frameworks and societal expectations related to gender roles can add depth to our insight into these relationships. Studies have indicated that societal influences can impact girls’ motivation to engage in certain sports or activities compared with boys. Hence, while chronological age provides a foundational perspective on physical development, it is essential to consider how sociocultural environments interact with age to shape children’s motor development experiences [5,7,10].

### Limitations and Directions for Further Research

While our results are generally consistent with similar studies [13,15,16], the cross-sectional design in this study and our small convenience sample limit inferences from these data and the generalizability of our study.

It was not practicable to use skeletal age to measure biological maturity in our sample of prepubertal children because of costs and radiological exposure. Noninvasive equation-based models such as the Khamis–Roche method are generally considered the most practical option for field studies that require estimating children’s biological maturation. The Khamis–Roche method has sound psychometric support; however, it is a prediction model and, therefore, has a degree of associated error that needs to be considered.

Overweight and obesity in particular are consistently negatively associated with children’s MC. For this reason, a measure of adiposity is necessary in studies assessing children’s MC. Although BMI is the standard measure for adiposity in field studies, it is a poor measure of body fat distribution. Alternate measures such as bioelectrical impedance analysis (BIA) will provide an accurate assessment of body composition, but BIA is not necessarily feasible in field studies involving young children (e.g., hydration levels, fasting, and restricting physical activity prior to measurement).

We did not measure environmental (social, physical), psychological, or cultural variables that might be associated with MC. Newell’s Model of Constraints posits that MC is a consequence of the interaction between the cognitive and physical characteristics of a person, the task performed, and the environment in which the task is performed [6]. Given that we found an association between age, but not biological maturation, and MC, there is reason to assume that a third or fourth set of variables of relevance to MC might be more closely related to age than maturation. For example, for young children the role of parents (e.g., parenting styles, health behaviors) and siblings in the home environment and peer and classroom interactions at school appear to have some influence on their motor development [42,43]. Currently, these variables have received less research attention on how they influence children’s MC, and these variables should be considered in larger and more comprehensive future studies of MC.

Future research should consider objective measures of physical activity and participation in organized and non-organized sports to further elucidate their role in MC. Previous research has found inconsistent evidence for the MC and physical activity pathway in particular, with inconclusive evidence for the path from physical activity to MC, no evidence for locomotor, coordination, and stability skills, and indeterminate evidence for object control skills. The evidence for the reverse pathway, MC to physical activity, was also indeterminate [3]. It has been suggested that individual differences in maturity may more strongly affect, positively or negatively, performance on health-related physical fitness tests than on MC and that the nature of the association may vary in relation to the age and sex of the individual and the nature and demands of the task. A network approach on longitudinal data may better capture the dynamic interplay among direct and indirect factors and how they influence MC across different developmental stages.

## 5. Conclusions

The current study examined the correlates of MC as a part of a complex network in which each variable has a dynamic role within the emerging pattern. Our results demonstrate that age, not biological maturation, was associated with MC in prepubertal boys and girls. The network analysis indicated that the catch, dribble, and run in boys and the gallop and slide in girls had higher centrality indices potentially reflecting activity preferences (e.g., ball games for boys, dance for girls). The findings of this study corroborate prior findings that, in prepubertal children, biological maturation seems to be negligibly related to MC, even while age was closely related.

## Figures and Tables

**Table 1:** Descriptive statistics (mean ± standard deviation) by sex and test for equality of means in addition to the mean difference, including the standard error, 95% confidence limits, and effect size (n = 218).

	Descriptive Statistics (Mean ± SD)		Mean Difference			Effect Size		Equality of Means	
Variables	Girls (n = 118)	Boys (n = 100)	Mean	SE	95% CL	Cohen’s d	(Qualitative)	t (df = 216)	*p*
Age (years)	8.12 ± 1.0	7.86 ± 0.9	0.254	0.130	(−0.02; 0.51)	0.26	(small)	1.954	0.052
PMS (cm)	163 ± 5.37	177.9 ± 6.09	−14.90	0.777	(−16.4; −13.3)	−2.59	(very large)	−19.160	<0.001 *
Attained PMS (%)	78.96 ± 4.35	72.83 ± 3.48	6.123	0.541	(5.05; 7.18)	1.55	(moderate)	11.310	<0.001 *
Maturity z-score	−0.57 ± 1.33	0.64 ± 1.55	−1.218	0.196	(−1.60; −0.83)	−0.84	(moderate)	−6.222	<0.001 *
Height (cm)	129.61 ± 8.27	128.67 ± 7.73	−0.939	1.086	(−3.07; 1.20)	−0.11	(trivial)	−0.865	0.388
Weight (kg)	29.43 ± 7.13	29.77 ± 9.26	−0.334	1.123	(−2.54; 1.88)	−0.04	(trivial)	−0.298	0.766
BMI (kg/m^2^)	17.60 ± 3.1	17.35 ± 3.6	0.525	0.463	(−0.66; 1.16)	0.07	(trivial)	0.525	0.587
Two-hand strike	7.76 ± 1.5	8.27 ± 1.4	−0.507	0.202	(−0.90; −0.10)	−0.34	(small)	−2.511	0.013 *
One-hand strike	4.92 ± 2.0	5.55 ± 2.0	−0.626	0.277	(−1.17; −0.08)	−0.30	(small)	−2.258	0.025 *
Dribble	4.86 ± 1.5	4.81 ± 1.6	0.054	0.213	(−0.36; 0.47)	0.03	(trivial)	0.256	0.798
Catch	5.28 ± 0.9	5.57 ± 0.8	−0.282	0.125	(−0.52; −0.03)	−0.30	(small)	−2.255	0.025 *
Kick	6.78 ± 1.4	6.64 ± 1.4	0.140	0.192	(−0.23; 0.51)	0.09	(trivial)	0.727	0.468
Over-hand throw	5.64 ± 1.9	6.44 ± 2.0	−0.796	0.266	(−1.30; −0.27)	−0.40	(small)	−2.991	0.003 *
Under-hand throw	6.68 ± 1.3	6.82 ± 1.5	−0.134	0.190	(−0.50; 0.24)	−0.09	(trivial)	−0.705	0.482
Run	7.75 ± 0.7	7.44 ± 0.9	0.314	0.116	(0.08; 0.54)	0.36	(small)	2.699	0.008 *
Gallop	7.40 ± 1.4	7.23 ± 1.1	0.177	0.176	(−0.17; 0.52)	0.13	(trivial)	1.002	0.318
Hop	7.17 ± 1.4	7.01 ± 1.3	0.168	0.185	(−0.19; 0.53)	0.12	(trivial)	0.906	0.366
Skip	5.08 ± 1.6	4.92 ± 1.8	0.165	0.237	(−0.30; 0.63)	0.09	(trivial)	0.695	0.488
Jump	6.82 ± 1.3	6.48 ± 1.5	0.342	0.196	(−0.04; 0.72)	0.23	(small)	1.743	0.083
Slide	7.71 ± 0.9	7.77 ± 0.8	−0.058	0.117	(−0.28; 0.17)	−0.06	(trivial)	−0.497	0.620

Note. SE, standard error; 95%CL, 95% confidence limits; d, d-Cohen for determining effect size; PMS, predicted mature stature; PMS%, percentage of predicted mature stature; BMI, body mass index.; * *p* < 0.05.

**Table 2:** Association between the study variables from the perspective of network analysis by sex.

Girls																	
Variables		1	2	3	4	5	6	7	8	9	10	11	12	13	14	15	16
1	Two-hand strike	0.000															
2	One-hand strike	0.000															
3	Dribble	0.000	0.000														
4	Catch	0.153	0.055	0.228													
5	Kick	0.000	0.000	0.132	0.016												
6	Over-hand throw	0.125	0.086	0.040	0.095	0.125											
7	Under-hand throw	0.087	0.000	0.000	0.118	0.000	0.182										
8	Run	0.008	0.000	0.015	0.038	0.088	0.124	0.000									
9	Gallop	0.019	0.057	0.000	0.033	0.000	0.000	0.056	0.000								
10	Hop	0.000	0.000	0.000	0.000	0.000	0.000	0.000	0.406	0.068							
11	Skip	0.000	0.000	0.043	0.000	0.000	0.000	0.027	0.000	0.177	0.134						
12	Jump	0.000	0.163	0.000	0.000	0.000	0.000	0.081	0.231	0.107	0.000	0.055					
13	Slide	0.101	0.186	−0.084	0.000	0.284	0.000	0.208	0.000	0.137	0.181	0.161	0.000				
14	Ch Age vr	0.000	0.000	0.169	0.138	0.000	0.108	0.037	0.000	0.297	0.000	0.000	0.000	0.000			
15	KR-PMS z-score	0.000	0.000	−0.039	0.000	0.000	0.000	0.038	0.000	0.064	0.000	0.000	0.000	0.000	0.000		
16	BMI	0.000	0.000	0.000	0.000	0.042	0.027	0.000	0.000	0.106	0.000	0.051	0.000	0.000	0.054	0.579	0.000
Boys																	
Variables		1	2	3	4	5	6	7	8	9	10	11	12	13	14	15	16
1	Two-hand strike																
2	One-hand strike	0.000															
3	Dribble	0.000	0.082														
4	Catch	0.153	0.000	0.000													
5	Kick	0.000	0.000	0.000	0.270												
6	Over-hand throw	0.125	0.042	0.084	0.000	0.000											
7	Under-hand throw	0.087	0.215	0.000	0.205	0.088											
8	Run	0.008	0.014	0.163	0.051	0.000	0.000	0.000									
9	Gallop	0.019	0.000	0.000	0.000	0.000	0.000	0.000	0.000								
10	Hop	0.000	0.000	0.000	0.000	0.000	0.000	0.000	0.000	0.061							
11	Skip	0.000	0.000	0.000	0.000	0.000	0.000	0.000	0.187	0.000	0.161						
12	Jump	0.000	0.000	0.064	0.000	0.000	0.000	0.000	0.111	0.000	0.000	0.000					
13	Slide	0.101	0.000	0.000	0.000	0.284	0.170	0.000	0.088	0.000	0.000	0.000	0.000				
14	Ch_Age_vr	0.000	0.000	0.074	0.068	0.000	0.000	0.000	0.022	0.000	0.000	0.000	0.000	0.000			
15	KR-PMS z-score	0.000	0.000	0.000	0.000	0.000	0.000	0.000	0.000	0.000	0.000	0.000	0.000	0.000	0.000	0.000	
16	BMI	0.000	0.000	0.069	0.000	0.042	0.000	0.000	0.000	0.000	−0.033	0.000	0.000	−0.058	0.134	0.267	0.000

Note. Ch-age-yr, chronological age; Biological maturation KR-PMS-z-score, Khamis and Roche predicted maturity status z-score; BMI, body mass index.

**Table 3:** Values of the network centrality indicators for the total sample and by sex.

Variables	Betweenness			Closeness			Strength			Expected Influence		
	All	Girls	Boys	All	Girls	Boys	All	Girls	Boys	All	Girls	Boys
Age	0.675 *	0.675 *	−9.333	0.546	0.546	−0.720	−0.020	−0.020	−0.653	0.133	0.133	−0.558
KR-PMS-z-score	−0.765	−0.765	−0.933	−2.000	−1.998	−1.084	−0.420	−0.420	−0.901	−0.676	−0.676	−0.558
BMI	0.495	0.495	0.400	−1.661	−1.661	−0.570	0.243	0.243	0.340	0.408	0.408	−0.336
Two-hand strike	−0.756	−0.756	−0.933	−0.910	−0.910	−1.199	−1.510	−1.505	−1.056	−1.421	−1.421	−0.963
One-hand strike	−0.585	−0.585	−0.933	−0.180	−0.180	−0.750	−1.240	−1.240	−0.540	−1.145	−1.145	−0.445
Dribble	−0.585	−0.585	1.822 *	−0.241	−0.241	1.540 *	−0.275	−0.275	1.629 *	−1.361	−1.361	1.730 *
Catch	−0.135	−0.135	0.933	−0.370	−0.370	1.156 *	0.314	0.314	1.870 *	0.482	0.482	1.972 *
Kick	−0.405	−0.405	−0.222	0.664 *	0.664 *	0.84	−0.577	−0.577	0.192	−0.451	−0.451	0.289
Over-hand throw	−0.405	−0.405	−0.756	0.287	0.287	0.258	0.493 *	0.493 *	−0.136	0.669 *	0.669 *	−0.039
Under-hand throw	−0.405	−0.405	−0.489	0.431	0.431	0.032	0.125	0.125	−0.207	0.284	0.284	−0.111
Run	−0.315	−0.315	1.200 *	−0.182	−0.182	1.285 *	0.491	0.491	1.232 *	0.667	0.667	1.332 *
Gallop	2.475 *	2.475 *	−0.933	1.300 *	1.300 *	−1.947	1.497 *	1.497 *	−1.755	1.719 *	1.719 *	−1.663
Hop	−0.404	−0.405	0.311	0.128	0.128	−0.058	−0.096	−0.096	−0.631	0.052	0.052	−0.819
Skip	−0.765	−0.765	1.467 *	0.402	0.402	0.917	−0.763	−0.763	−0.468	−0.646	−0.646	0.566
Jump	−0.315	−0.315	−0.933	−0.273	−0.273	−0.144	−0.809	−0.809	−0.542	−0.694	−0.694	−0.447
Slide	2.205 *	2.205 *	0.933	2.053 *	2.053 *	0.445	2.543 *	2.543 *	0.691	1.978 *	1.978 *	0.299

Note. KR-PMS-z-score, Khamis and Roche predicted maturity status z-score; BMI, body mass index. * Highlighted variables in the model.

## Data Availability

The data presented in this study are available on request from the corresponding author. The data are not publicly available due to restrictions, e.g., privacy or ethical.
